# The Association between Temperament, Chronotype, Depressive Symptoms, and Disease Activity among Patients with Inflammatory Bowel Disease—A Cross-Sectional Pilot Study

**DOI:** 10.3390/life11121347

**Published:** 2021-12-05

**Authors:** Łukasz Mokros, Danuta Domżał-Magrowska, Tadeusz Pietras, Kasper Sipowicz, Renata Talar-Wojnarowska

**Affiliations:** 1 Department of Clinical Pharmacology, Medical University of Łódź, Kopcińskiego 22, 90-153 Łódź, Poland; 2 Department of Digestive Tract Diseases, Medical University of Lodz, 90-153 Łódź, Poland; danuta.magrowska@gmail.com (D.D.-M.); r-wojnarowska@wp.pl (R.T.-W.); 3 Institute of Psychiatry and Neurology in Warsaw, Second Department of Psychiatry, Sobieskiego 9, 02-957 Warsaw, Poland; tpietras@ipin.edu.pl; 4 Department of Interdisciplinary Disability Studies, The Maria Grzegorzewska University in Warsaw, Szczesliwicka 40, 02-353 Warsaw, Poland; ksipowicz@aps.edu.pl

**Keywords:** Crohn’s disease, ulcerative colitis, depression, perseverance

## Abstract

The psychological aspect may play an important role in ulcerative colitis (UC) and Crohn’s disease (CD). The aims of this study were to explore the differences between patients with UC and CD regarding chronotype, temperament and depression, and to assess the psychological factors mentioned as predictors of disease activity. In total, *n* = 37 patients with UC and *n* = 47 patients with CD were included in the study. They underwent a clinical assessment, including the Mayo score or Crohn Disease Activity Index (CDAI), and completed questionnaires: a sociodemographic survey, Formal Characteristics of Behavior–Temperament Inventory (FCB-TI), Chronotype Questionnaire (CQ), and the Beck Depression Index II (BDI). The Sensory Sensitivity score was higher among patients with CD than UC (*p* = 0.04). The emotional reactivity and endurance scores were higher among women than men with CD (*p* = 0.028 and *p* = 0.012 respectively). CQ Morningness–Eveningness (ME) correlated with Endurance (*p* = 0.041), Emotional Reactivity (*p* = 0.016), and Activity (*p* = 0.004). ME correlated with Rhythmicity among CD patients (*p* = 0.002). The Mayo score was predicted by Perseverance. The CDAI score was predicted by the BDI score. The pattern of the relationship between chronotype and temperament may differentiate patients with UC and CD. Personal disposition may play a role in the clinical assessment of patients with IBD.

## 1. Introduction

Inflammatory bowel diseases (IBDs) are chronic conditions characterized by inflammation in different parts of the gastrointestinal tract with a multifactorial etiology, including genetic susceptibility, environmental factors, and the involvement of autoimmune mechanisms. Their most common forms are ulcerative colitis (UC) and Crohn’s disease (CD) [1,2]. Despite important clinical distinctions as well as diagnostic and therapeutic considerations, both diseases manifest in abdominal pain, diarrhea or fatigue—all of a remittent and recurrent character. Thus, IBDs seriously hamper patients’ quality of life, although life-threatening complications, e.g., bowel perforation, may occur as well [1,2]. The psychological burden of UC and CD may result in other mental health consequences. Patients with IBD have been shown to have higher indices of anxiety and depression compared to the healthy population; also, the severity of pain has been linked to greater risk of depression [3]. In fact, the risk of clinically significant depression may be particularly increased within the first year of IBD diagnosis [4]. Inversely, elevated depressive symptoms have been associated with increased odds of active IBD and the use of biological therapy in a three-year observation [5]. Poor sleep quality and insomnia also appear to be highly prevalent and linked to disease activity and depression among IBD patients [6].

Apart from the above-mentioned effects of IBD on well-being, certain temperamental features have been described as risk factors for CD and UC. Temperament is defined as a set of biologically determined, relatively fixed dispositions of an individual to react in a certain manner in certain situations [7]. High neuroticism (or emotional liability), impulsivity (i.e., inability to postpone reward), and alexithymia (impaired insight into one’s emotions) have been associated with the diagnosis of IBD [8,9]. Individual psychological differences also appear to be associated with the course of IBD. An increase in harm avoidance (a temperamental feature) has been negatively associated with IBD-related quality of life [10]. Maladaptive stress coping strategies (i.e., contributing to a global increase in distress instead of alleviating it, e.g., avoidance, rumination, escape) have been linked to the active phase of IBD, poor quality of life, depression, and anxiety [11,12]. In addition, IBD activity has been shown to correlate positively with cyclothymic, depressive, and anxious temperamental traits and negatively with hyperthymic temperament [13]. However, none of the studies to date have explored the link between the course of IBD and temperament in the light of the Regulative Temperament Theory of Strelau, which is one of the most renowned theories of temperament [14,15]. The search for the link between personal dispositions and morbidity stems from a paradigm that states that temperament (i.e., a relatively fixed disposition to react in a certain manner in specific situations) is biologically determined [16,17].

Chronotype, which is a personal disposition toward a certain circadian pattern of functioning, is also considered a temperamental feature [18]. Its basic component is morningness–eveningness, i.e., a preference regarding the optimal time of the day for activity; however, some studies suggest this has a multidimensional structure [19,20,21]. The evening chronotype, as a predisposition to a desynchronization between social cues and biological clock, has been repeatedly reported to be a risk factor for depression and suicidality as well as somatic diseases such as type II diabetes or asthma [22,23,24,25,26].

Regarding the gastrointestinal tract, it is indicated that many of its functions undergo circadian variability, including cell proliferation, motility, hormone secretion, absorption, microbiome, permeability, or immunity [27]. Consequently, an association between evening chronotype and an increased probability of gastrointestinal diseases has been found [28].

In the case of IBD patients, irregular eating and evening chronotype have been linked to poor quality of life [29]. The late type has also been associated with increased mental and physical fatigue and decreased daily activity [30].

Given the considerations mentioned, IBDs are determined by biopsychosocial factors. Thus, a search for disease activity correlates among temperamental traits appears to be valid. In addition, personality (i.e., individual psychological differences) may be a part of the clinical differentiation between UC and CD, as suggested by previous studies [13].

The aims of this study were (1) to explore whether there are differences between patients with UC and CD regarding chronotype, temperament, and depression, and (2) to assess the link between the psychological factors mentioned above and IBD activity.

## 2. Materials and Methods

### 2.1. Study Design and Sample

This was a single-center, cross-sectional study, conducted from 5 December 2019 to 6 August 2020 on a non-random sample of patients of the Gastroenterology Outpatient Clinic and the Clinical Ward of the Department of Digestive Tract Diseases at the Barlicki Memorial Clinical Hospital no. 1 in Lodz, Poland. The inclusion criteria were age of at least 18, a confirmed diagnosis of ulcerative colitis or Crohn’s disease, and informed consent for participation in the study. The exclusion criteria were lack of informed consent, diagnosis of major neurocognitive disorder, diagnosis of cancer, serious and unstable heart disease, a severe psychological trauma within the six months preceding the study, and serious mental illness (e.g., schizophrenia or bipolar affective disorder or other psychotic disorder).

In total, 87 sets of questionnaires were handed out personally by the researchers (DDM, RTW) to patients upon their routine visit to the outpatient clinic or during their hospitalization. After signing the informed consent form, the participants underwent a structured interview, which was conducted by a researcher, and they were asked to complete a set of self-report questionnaires, with an option of asking for assistance from the researcher at any point. All patients returned the forms. The clinical and demographic data including sex, age, the course of IBD, and comorbidities were collected on the basis of the interview and recent medical documentation. Upon completion of the questionnaires and the interview, the patients were once again checked by a researcher for compliance with the exclusion/inclusion criteria and the completeness of their forms. At this point, three patients were excluded. Therefore, the final sample for the preliminary analysis comprised *n* = 37 patients with UC and *n* = 47 with CD.

### 2.2. Questionnaires

The questionnaires used were all recognized and sufficient, in terms of validity and reliability, for use in clinical practice, psychological diagnosis (in the case of psychological tests), and scientific research.

The Mayo Score is an UC activity index created by Schroeder et al. and is utilized to assess the severity of the disease, mainly for the purposes of the clinical trials [31,32,33]. It consists of four items considering stool frequency, rectal bleedings, mucosal appearance in endoscopy (in this study, the item was completed based on recent colonoscopy results), and general assessment of the disease activity by the physician. The score ranges from 0 to 12, with a higher number indicating a higher level of disease activity. Although no formal validation of the scale was performed, the Mayo score has been repeatedly reported to correlate with UC activity [32,33].

The Crohn’s Disease Activity Index (CDAI) was developed by Best et al. [34]. The scale consists of eight questions on the recent clinical status of the patient, blood hematocrit (in this study, the item was completed on the basis of a recent complete blood count), and the general well-being of the patient. The total score is calculated on the basis of the algorithm developed. The higher the score, the greater the level of disease activity. Theoretically, a maximum score depends on the number of complaints and cannot be delineated, but a score of over 450 marks severe CD [34,35,36].

The Functional Characteristics of Behavior–Temperament Inventory Revised (FCB-TI) was utilized to measure temperament according to the Regulative Temperament Theory of Strelau [7]. The revised version of the questionnaire was devised and validated by Cyniak-Cieciura, Zawadzki, and Strelau [37]. The questionnaire consists of seven scales: four representing the energetic aspect of behavior (Sensory Sensitivity, Endurance, Emotional Reactivity, Activity) and three representing the temporal characteristics of behavior (Briskness, Perseverance, Rhythmicity) (Table 1). Each scale comprises 15 items, and their total score ranges from 15 to 60—the higher the score, the greater the intensity of the trait. Cronbach’s alpha values for the scales ranged from 0.76 to 0.86 in the normalization study [37].

The Chronotype Questionnaire (CQ) was constructed and validated by Oginska et al. [19]. It comprises two dimensions: Morningness–Eveningness (ME) and the subjective amplitude, or distinctness, of the rhythm (AM). Higher ME scores indicate a greater preference for morning activity. AM is defined as the ability to sense or adjust activity levels depending on the time of the day. A higher score indicates a greater amplitude. The ME scale consists of eight items, while the AM comprises six, with possible scores ranging from 8 to 40 for the ME and 6 to 30 for the AM. Cronbach’s alpha values are 0.84 for the ME scale and 0.77 for the AM scale [19].

The Beck Depression Inventory version II (BDI) was utilized to assess the severity of depressive symptoms. The scale was adapted to Polish and validated by Zawadzki et al. [38,39]. The test consists of twenty-one items considering the occurrence and intensity of depressive symptoms within the previous two weeks. Each item is scored from 0 to 3, which gives a possible range of 0 to 63 points. The higher the score, the greater the severity of depression. The Cronbach’s alpha of the Polish version of the questionnaire was 0.93 among patients with depressive disorder [38,39].

### 2.3. Statistical Analysis

The data were analyzed in STATISTICA version 13 (Dell, the United States). The categorical variables were characterized as the number of observations and percentages. The continuous variables were characterized by their minimal–maximal range and mean values with standard deviations (SD). The normality of distribution was verified with the Shapiro–Wilk W test and by an analysis of histograms. Intergroup comparisons were conducted via the analysis of variance (ANOVA) or with Welch’s *t*-test, depending on whether the assumption of homogeneity of variance was met. The heterogeneity of variance between the subgroups was checked with Levene’s test. ANOVA was performed in 2 × 2 groups, which were established by sex and disease. Thus, three effects were tested: sex, disease, and sex * disease. In cases of statistically significant intergroup differences, Tukey’s post hoc test was applied.

An association between two continuous variables was initially assessed with Pearson’s correlation quotient or Spearman’s rank correlation quotient, depending on whether normal or non-normal distribution was assumed, respectively. Selected correlation quotients were compared pairwise, and the probability in the test was reported in the text. The correlation quotients may be interpreted in terms of Cohen’s thresholds for small (0.1), medium (0.3), and strong size of effect (0.5) [40].

Based on the analysis of statistically significant correlates of CDAI and the Mayo scores, the psychological variables were chosen for linear regression models, separately for each disease severity score. Both models were adjusted for the age and sex of the patient. The qualitative variables were coded with sigma restrictions. The tolerance indices were analyzed to track possible multicollinearities. Bootstrapping, with a sampling set at *n* = 1000, was performed to empower the results of the linear regression models and account for possible non-parametric distribution. Statistical significance was defined as *p* < 0.05 or a confidence interval not encompassing 0. A post hoc power estimation was performed for each analysis, with an assumption that this was an exploratory, preliminary analysis.

### 2.4. Data Availability Statement

The database used to support this study’s findings may be obtained upon request submitted to the corresponding author.

### 2.5. Ethics Statement

This study was conducted in accordance with the Declaration of Helsinki. The study protocol was approved by the Bioethical Committee of the Medical University of Lodz (RNN/10/20/KE, 14 January 2020).

## 3. Results

### 3.1. Intergroup Comparisons

There was a statistically significant difference in age and the scores of Sensory Sensitivity between the disease groups: patients with CD were on average younger (*p* = 0.018) and had higher Sensory Sensitivity (*p* = 0.04) than patients with UC. Those differences were not confirmed in the post hoc analysis.

In addition, a statistically significant difference was seen in the scores of CQ Amplitude of the Rhythm, Endurance, Emotional Reactivity, Briskness, and Perseverance between all men and all women in the studied group. Women had on average a higher subjective amplitude of the Rhythm (*p* = 0.023), lower Endurance (*p* = 0.025), higher Emotional Reactivity (*p* = 0.007), lower Briskness (*p* = 0.016), and higher Perseverance (*p* = 0.021) than men. Yet, a post hoc analysis revealed that it concerned only patients with CD: men had higher Endurance scores (*p* = 0.028) and lower Emotional Reactivity (*p* = 0.012) scores than women. No sex * disease was found statistically significant in the conducted analysis. Yet, the difference regarding the BDI score between the groups delineated by sex * disease interaction was on the verge of statistical significance (*p* = 0.065) (Table 2).

### 3.2. Correlations

In this subsection, only selected statistically significant correlations are characterized. A detailed correlation quotient matrix is presented in Table 3.

Among both patients with UC and CD, the Subjective amplitude of the rhythm correlated negatively with Endurance (*p* = 0.003 and *p* < 0.001, respectively) and with Briskness (*p* ≤ 0.001 and *p* = 0.045), yet the latter was stronger in the UC than the CD group (*p* < 0.05). In both groups, Endurance correlated with Emotional Reactivity (*p* = 0.001 and *p* < 0.001), Activity (*p* < 0.001 and *p* < 0.001), and Briskness (*p* < 0.001 and *p* < 0.001); Emotional Reactivity correlated with Briskness (*p* = 0.005 and *p* < 0.001), Perseverance (*p* = 0.002 and *p* < 0.001), and BDI score (*p* = 0.002 and *p* < 0.001). In addition, BDI score correlated negatively with Endurance (*p* = 0.006 and *p* = 0.001), Activity (*p* = 0.001 and *p* < 0.001), and Briskness (*p* < 0.001 and *p* < 0.001).

Only among patients with UC did Morningness–Eveningness correlate with Endurance (*p* = 0.041), Emotional Reactivity (a negative correlation, *p* = 0.016), and Activity (*p* = 0.004), while among CD patients, there was a negative correlation between Morningness–Eveningness and Rhythmicity (*p* = 0.005). Regarding the Subjective amplitude of the rhythm, there were positive correlations with Emotional Reactivity (*p* = 0.02) and Rhythmicity (*p* = 0.042) and a negative one with Activity (*p* = 0.027) among UC patients. There was a negative correlation between Briskness and Rhythmicity (*p* = 0.044) but only among UC patients.

Among UC patients, the Mayo severity score correlated negatively with Activity (*p* = 0.013) and Perseverance (*p* = 0.001), while among CD patients, the CDAI score correlated only with the BDI score (*p* = 0.017) (Table 3).

### 3.3. Linear Regression Models

The linear regression model predicting the Mayo Disease Severity score was fit to the empirical data. In the model, the association between the Mayo score and Perseverance, but not Activity, was confirmed. Perseverance was negatively associated with the Mayo score independently of a patient’s age and sex (Table 4).

The linear regression model predicting the Crohn’s Disease Activity Index score was fit to the empirical data. In the model, the association between the CDAI and BDI scores was confirmed. A rise in depression was linked to an increase in the CDAI score independently of the patient’s age and sex (Table 4).

## 4. Discussion

This is one of the first studies investigating temperament among adult patients with IBDs, and certain important findings can be delineated. In this study, patients with CD had on average higher Sensory Sensitivity compared to UC patients. High Sensory Sensitivity means having a low threshold for different stimuli, which may include subtle sounds, smells, but also probably subtle signs of the recurrence of the active phase of the disease. However, this statement is highly speculative and based merely on the description of the Sensory Sensitivity scale, since no comparable studies were published [37]. In addition, in the case of CD patients, men had higher Endurance scores and lower Emotional Reactivity scores compared to women. This association reflects a general tendency regarding the sex differences in temperamental structure between men and women seen in the healthy population [37]. It may raise a question as to why such a relationship was not statistically significant among the UC patients. The discrepancy may be due to either a too-small sample or a difference in mean age between UC and CD groups. Bielinski et al. found a greater severity of cyclothymic, depressive, and irritable affective temperaments among CD patients compared with UC patients [13]. Vidal et al. found no difference in harm avoidance, novelty seeking, reward dependence, and persistence (i.e., psychobiological temperamental features) between patients with active vs. remitted IBD [41]. It should be noted that those studies employed different methodological and theoretical backgrounds for the assessment of temperament, so the value of a comparison between those studies and this research is limited.

Notably, the patterns of relationship between chronotype and temperament were different for UC and CD patients. Jankowski found that a tendency toward morningness was associated with a rise in Endurance and a fall in Emotional Reactivity [42]. Similar, yet stronger, associations were found among UC but not CD patients in the current study. In addition, the link (of a moderate effect size) between evening preference and increased depressive symptoms was seen among UC but not CD patients. It is widely recognized that the late chronotype is a risk factor for depression in the general population [22]. Based on those premises, it may be suspected that the pattern of relationship between chronotype, temperament, and depressive symptoms among patients with UC may have more in common with the pattern seen in the healthy population than in patients suffering from CD. Those discrepancies might be an important part of the differentiation between the types of IBDs [13]. However, given the limited evidence and small sample in this study, this statement is merely speculative, and further research is required.

The analysis of the correlations in disease activity provides additional information. Among patients with UC, disease activity correlated negatively with two temperamental traits, namely Activity and Perseverance, but only the latter was confirmed in a linear regression, which was adjusted for sex and age. This would mean that low Perseverance (i.e., a tendency to easily forget about decisions made, past experiences, and absence of a tendency to repeat certain behaviors in stressful situations) predicted greater UC activity. The only similar study reported on no statistically significant association between temperament and disease activity in UC [13]. However, the current results appear to be plausible in the light of previous research. By definition, low Perseverance may mean poor adherence to a physician’s recommendations [7,37]. In the case of UC, adherence and perseverance in treatment have been proven to be crucial in avoiding exacerbations of the disease [43]. Non-adherence to treatment comes with a psychological burden, including increased perceived stress [44]. Interestingly, the association between depression and activity in UC described in previous studies was not found to be significant in the current study [45].

Regarding the CD patients, only the severity of depressive symptoms (from among the selected psychological variables) was found to predict the activity of the disease. Similarly to Bielinski et al., no statistically significant association between temperamental features and disease activity in patients with CD was observed [13]. This model replicated the results of previous studies associating the increased severity of depression with a more aggressive course of CD [45,46]. In the light of the so-called inflammatory theory of depression, the immune activation in CD flares may provide an explanation for the observed association [47]. These results confirm the previous findings that clinically significant depressive symptoms are linked to poor prognosis in CD [45,48]. It may raise concern as to why no similar association was found among UC patients in the current study, since depression was recently found to substantially increase the risk of active disease both in UC and CD [5]. It should be noted that there were several methodological discrepancies between the study presented and that by Marrie et al., including time of observation, size of sample, and questionnaires used (assessing both depression and disease activity) [5]. Thus, further research is warranted.

In the current study, the association between chronotype and disease activity in IBD was not found to be significant. Nevertheless, the topic requires further research, since chronotype was recently associated with nutrition and clinical parameters among IBD patients [49]. For example, evening types exhibited higher glucose and magnesium serum levels and lower iron and hematocrit levels compared to morning types, which may have consequences for the disease activity [49].

A practical aspect can be fostered based on these results. CD and UC are considered psychosomatic diseases determined by biopsychosocial factors. Thus, IBDs require multidisciplinary care [50,51]. It appears that the prevalence of depressive and anxiety symptoms is similar between IBDs and other chronic somatic diseases [52]. Yet, the assessment of depressive symptoms should probably be considered as a standard element of disease activity evaluation, since the relationship appears to be bidirectional [5]. Taking into account the temperamental traits may help in providing patient-tailored therapeutic interventions and increase their effectiveness [50]. This appears important, since the history of patients’ therapy has been associated with their psychosocial functioning [53]. Considerations for personal dispositions regarding the course and therapy of IBDs stays in line with the precision medicine paradigm [54].

### Limitations

Certain limitations should be noted regarding the current study’s design and the implications of the interpretation of its results. The sample was small, non-random, and originated from a single center. In addition, there was a difference in mean age between the UC and CD groups. These aspects hamper the generalizability of the findings. However, the sample represented a naturalistic, clinical group. The large number of statistical tests performed posed a risk of type I error, yet it should be noted that the analysis had an exploratory character and that certain corrections were abandoned. At the same time, regression models underwent bootstrapping with *n* = 1000 sampling to empower the results. Another limitation is the observational, single time-point character of the study. In addition, it did not include certain clinical variables, such as current pharmacological treatment. A study with a prospective, multicenter design, including a larger number of IBD patients, might be of particular value in examining the predictive role of temperament and chronotype in disease activity. It should be noted that previous studies employed different methodological and theoretical backgrounds for the assessment of temperament, so the value of a comparison between those and the research presented is limited. In a larger study group, a deepened analysis of the types of temperament, as delineated by Strelau, would also be possible and would probably provide additional knowledge on the complex association between personal dispositions and the course of IBDs. Only self-reported assessment measures were utilized. Notably, the questionnaires chosen for the study are widely recognized and highly valued tools used in clinical practice and scientific research. In addition, the regression models constructed in this study were adjusted for both sex and age to minimize the effect of bias of those two variables. It should also be raised that the study period of the current research encompassed the first lockdown due to the coronavirus disease 2019 (COVID-19) outbreak on 16 March 2020. Yet, since a certain proportion of the patients were already examined and personal dispositions (not situational psychosocial matters) were main variables of interest, the primary study protocol was kept. However, previous literature indicated that the COVID-19 outbreak and related lifestyle changes may have influenced the quality of life and disease activity among IBD patients, yet the contribution of the lockdown-related factors appeared to be relatively small [55].

## 5. Conclusions

Patients with CD exhibited higher sensory sensitivity compared to UC patients. The pattern of the relationship between chronotype and temperament may differentiate patients with UC and CD. Low perseverance may predict an increase in disease activity in UC. A rise in the severity of depressive symptoms may predict a rise in disease activity in CD. The assessment of personal dispositions and depression among patients with IBD may provide significant therapeutic and prognostic findings. However, these conclusions should be interpreted with caution, as this was a cross-sectional study with a small sample size. A longitudinal study is warranted to better explore these relationships.

## Figures and Tables

**Table 1:** Descriptions of the temperamental features according to The Functional Characteristics of Behavior, based on their original definitions [37].

Name	Definition
Sensory Sensitivity	The ability to detect or react to subtle stimuli or subtle changes in stimuli, e.g., tactile, olfactory, gustatory, visual, or auditory.
Endurance	The ability to react adequately to prolonged and intensive stimuli—reflects the resistance to distractors and fatigue.
Emotional Reactivity	A propensity to intensive, emotional reactions—linked to emotional sensitivity and liability.
Activity	A tendency to engage highly stimulating behaviors.
Briskness	A tendency to fast reactions and maintenance of high speed of activities—reflects high flexibility to the change of situational conditions.
Perseverance	A propensity to continue or repeat certain behaviors after the end of the responsible stimuli.
Rhythmicity	A tendency toward a regular pattern of the circadian rhythm, including the sleep–wake cycle and time of eating.

**Table 2:** Comparison of the variables of interest between patients diagnosed with ulcerative colitis and Crohn’s disease, divided by sex, in the studied sample.

	Ulcerative Colitis				Crohn’s Disease						
	Women (*n* = 19)		Men (*n* = 18)		Women (*n* = 25)		Men (*n* = 22)				
	M	SD	M	SD	M	SD	M	SD	*p* (Sex)	*p* (Disease)	*p* (Sex * Disease)
Age	42.01	15.95	43.27	15.38	33.95	11.82	37.26	9.67	0.433	0.018	0.724
ME	19.32	5.43	19.72	4.64	20.20	5.92	21.09	5.26	0.586	0.345	0.839
AM	22.05	3.39	20.39	4.42	21.44	3.62	19.05	4.42	0.023	0.267	0.677
Sensory Sensitivity	41.68	5.94	40.33	5.34	42.80	4.00	44.09	5.94	0.980	0.040	0.260
Endurance	32.95	4.74	33.89	5.48	31.80	5.60	37.27 †	8.65	0.025	0.427	0.110
Emotional Reactivity	40.84	8.79	38.94	6.78	42.48	7.29	35.59 †	5.89	0.007	0.591	0.120
Activity	36.74	7.41	37.28	12.02	36.64	7.38	38.64	6.64	0.496	0.734	0.696
Briskness	40.74	4.51	43.11	8.11	39.48	6.65	44.05	6.01	0.016	0.910	0.441
Perseverance	42.68	4.92	41.33	5.01	43.64	5.18	39.73	5.09	0.021	0.771	0.254
Rhythmicity	23.63	4.47	24.44	7.25	24.48	4.91	22.09	4.67	0.505	0.525	0.178
BDI	9.84	9.53	11.17	9.67	17.12	11.29	10.50	7.40	0.216	0.123	0.065
	**Me**	**Q1–Q3**	**Me**	**Q1–Q3**	**Me**	**Q1–Q3**	**Me**	**Q1–Q3**	***p***		
MAYO	4.00	0.00–5.00	4.00	1.50–8.50					0.488		
CDAI					129.00	112.00–242.00	71.00	40.00–145.00	0.040		

ME—Morningness–Eveningness, AM—subjective amplitude of the circadian rhythm, BDI—Beck Depression Inventory II, Mayo—The Mayo severity score of the ulcerative colitis, CDAI—Crohn’s disease assessment index, N—number of observations, M—mean value, SD—standard deviation, p—probability in the respective test, Me—median value, Q1–Q3—range between first and third quartile, †—*p* < 0.05 vs. women with Crohn’s disease in post hoc test.

**Table 3:** Matrix of correlation quotients between the selected variables (i.e., chronotype, temperament traits, depression, severity of the disease) among patients with ulcerative colitis and Crohn’s disease in the studied sample.

Ulcerative Colitis Patients										
	ME	AM	Sens	Endur	Emot	Act	Brisk	Persev	Rhythm	BDI
**AM**	−0.043									
**Sens**	0.088	−0.235								
**Endur**	0.338 *	−0.469 **	−0.300							
**Emot**	−0.394 *	0.381 *	−0.185	−0.509 †						
**Act**	0.462 **	−0.363 *	−0.139	0.568 †	−0.265					
**Brisk**	0.321	−0.644 †	0.169	0.546 †	−0.454 **	0.413 *				
**Persv**	−0.022	0.163	−0.047	−0.251	0.493 **	0.060	−0.189			
**Rhythm**	−0.148	0.336 *	0.067	−0.138	0.145	0.042	−0.413 **	−0.218		
**BDI**	−0.325 *	0.415 *	−0.069	−0.445 **	0.491 **	−0.518 †	−0.546 †	0.201	−0.028	
**Mayo**	−0.153	−0.001	0.226	−0.055	−0.106	−0.428 *	0.191	−0.531 **	0.126	0.050
**Crohn’s disease patients**										
	**ME**	**AM**	**Sens**	**Endur**	**Emot**	**Act**	**Brisk**	**Persev**	**Rhythm**	**BDI**
**AM**	0.160									
**Sens**	0.078	−0.294 *								
**Endur**	0.051	−0.496 †	0.189							
**Emot**	−0.257	0.224	−0.294 *	−0.554 †						
**Act**	0.135	−0.176	0.116	0.376 **	−0.142					
**Brisk**	−0.088	−0.293 *	0.239	0.406 **	−0.464 **	0.140				
**Persv**	−0.032	0.165	0.175	−0.302 *	0.545 †	−0.003	−0.359 *			
**Rhythm**	−0.439 **	−0.127	−0.344 *	−0.200	0.313 *	−0.240	−0.049	−0.021		
**BDI**	−0.047	0.330 *	−0.098	−0.366 *	0.551 †	−0.343 **	−0.597 †	0.528 †	0.064	
**CDAI**	0.045	0.194	−0.040	0.029	0.154	−0.261	−0.046	0.190	−0.054	0.420 *

ME—Morningness–Eveningness, AM—subjective amplitude rhythm, Sens—Sensory Sensitivity, Endur—Endurance, Emot—Emotional Reactivity, Act—Activity, Brisk—Briskness, Persev—Perseverance, Rhythm—Rythmicity, BDI—Beck Depression Inventory, Mayo—The Mayo severity score of the ulcerative colitis, CDAI—Crohn’s disease assessment index. For associations with Mayo and CDAI scores—Spearman’s rank correlation quotients are presented. The remaining are Pearson’s correlation quotients. *—*p* < 0.05, **—*p* < 0.01, †—*p* < 0.001 (all tests were two-sided). The correlation quotients which were statistically significant in both UC and CD are marked with the bold font.

**Table 4:** A summary of linear regression models predicting the Mayo Disease Severity (Mayo) score among ulcerative colitis patients and the Crohn’s Disease Activity Index (CDAI) score among patients with Crohn’s Disease in the studied sample. Presented as unstandardized mean values of the unstandardized parameters (B) and models’ coefficients of determination (R^2^), with respective 95% Confidence Intervals, as derived from bootstrapping with *n* = 1000 samples.

	Prediction of the Mayo Score		
	Parameter	95%CI	
Intercept	25.443	14.458	35.389
Age	−0.002	−0.089	0.079
Activity	−0.154	−0.258	0.003
Perseverance	−0.368 *	−0.575	−0.077
Male sex	0.043	−1.250	1.193
R^2^	0.447 *	0.146	0.537
	**Prediction of the CDAI Score**		
	**parameter**	**95%CI**	
Intercept	51.874	−88.311	154.496
Age	0.818	−2.280	4.660
BDI	3.431 *	0.684	8.679
Male sex	−23.068	−61.130	19.779
R^2^	0.319 *	0.046	0.440

R^2^—coefficient of determination of the model, * statistically significant, based on the values of 95% Confidence Intervals (CI).

## Data Availability

The database used to support this study’s findings may be obtained upon request to the corresponding author.
